# Pregnancy in the Setting of Asymptomatic Non-Cirrhotic Chronic Portal Vein Thrombosis Complicated by Pre-Eclampsia

**DOI:** 10.1155/2013/984271

**Published:** 2013-05-28

**Authors:** Işık Üstüner, Remzi Adnan Akdoğan, Emine Seda Güvendağ Güven, Figen Kır Şahin, Şenol Şentürk, Elif Akdoğan, Filiz Taşçı

**Affiliations:** ^1^Department of Obstetrics and Gynecology, Faculty of Medicine, Recep Tayyip Erdoğan University, Merkez, 53100 Rize, Turkey; ^2^Department of Gastroenterology, Faculty of Medicine, Recep Tayyip Erdoğan University, Merkez, 53100 Rize, Turkey; ^3^Department of Hematology, Faculty of Medicine, Recep Tayyip Erdoğan University, Merkez, 53100 Rize, Turkey; ^4^Department of Radiology, Faculty of Medicine, Recep Tayyip Erdoğan University, Merkez, 53100 Rize, Turkey

## Abstract

Portal vein thrombosis (PVT) can be chronic or acute in nature; it is characterized by a thrombus formation in the main portal vein and/or its right or left branches. Herein, we present a 36-year-old woman with asymptomatic noncirrhotic chronic PVT who developed preeclampsia in the later stage of pregnancy. This report will emphasize the clinical differential diagnosis, outcome, and management of pregnancies complicated by noncirrhotic PVT.

## 1. Introduction

Portal vein thrombosis (PVT) can be chronic or acute in nature; it is characterized by thrombus formation in the main portal vein and/or its right or left branches. The prevalence according to the population-based necropsy studies is about 1%, often secondary to cirrhosis or malignancy [1, 2]. Noncirrhotic PVT is extremely rare [3]. Various local and systemic risk factors are associated with adult-onset PVT including abdominal inflammation, infections, surgery, myeloproliferative disorders, congenital or acquired thrombophilia, obesity, oral contraceptive intake, pregnancy, and postpartum period [1, 2]. The extension of PVT to the mesenteric venous arches carries a high risk of the intestinal infarction, a serious complication with a reported mortality rate of 20–60% [1, 3].

Chronic PVT is characterized either by the rapid development of portoportal collaterals around the thrombosed portal vein which bypasses the obstructed venous segment or by a thin, contracted, and recanalized portal vein measuring less than 8 mm in diameter. Collectively porto-portal collaterals are given the name “portal cavernoma” [1]. Chronic PVT can be asymptomatic or may present with a portal cavernoma, portal hypertension, cholestasis, splenomegaly, ascites, gastroesophageal varices, and pancytopenia [4]. Fatal gastrointestinal bleeding due to portal hypertension or subclinical hepatic encephalopathy due to massive portosystemic shunting, recurrent thrombosis, and portal biliopathy (deformation of the biliary lumen) are some of the rare but feared complications of chronic PVT [1].

Herein we present a 36-year-old woman with asymptomatic chronic PVT who developed preeclampsia in the later stage of her pregnancy. The report will emphasize the clinical differential diagnosis, outcome, and management of pregnancies complicated by noncirrhotic PVT.

## 2. Case Report

A 36-year-old pregnant gravida 4 parity 1 woman at 38 weeks of gestation presented to the obstetrics clinic with right upper quadrant pain, uterine cramps, and headache. Obstetric history revealed that she had no definite follow-up or antenatal visits regarding her pregnancy. She only reported a formal clinical examination at 20 weeks of pregnancy in another clinic in which she had a pelvic examination and was told that she was progressing normally. She had a healthy term delivery 14 years ago in which Cesarean section (C/S) was employed due to fetal stress. She also revealed that two years ago she presented to the gastroenterology clinic of another medical facility due to repeated episodes of abdominal pain. The woman had been told that she had a disease involving the liver and the biliary system but she was incompliant and did not pursue her follow-up appointments because she felt better. 

Her physical examination revealed a high arterial blood pressure (160/100 mm Hg) but her other vital signs were normal. No cervical dilatation or effacement was noted in the obstetric examination; however, she had edema and abdominal distention. Uterine tocography showed regular uterine contractions. Laboratory studies showed proteinuria (+1 by stick); biochemistry showed mild to moderate anemia (Hb: 10,3 g/dL), thrombocytopenia (64.5 K/uL), and hypoalbuminemia (2.6 mg/dL). The results of the renal and liver function tests were within normal limits. She was diagnosed with preeclampsia and was started on magnesium sulfate treatment. A crossmatch was undertaken and arrangements were made for prospective blood transfusions. An emergent C/S delivery under general anesthesia was carried out and a 2700 g healthy male infant with Apgar scores of 8 and 9 at 1 min, and 5 min, respectively was delivered.

**Figure 1:**
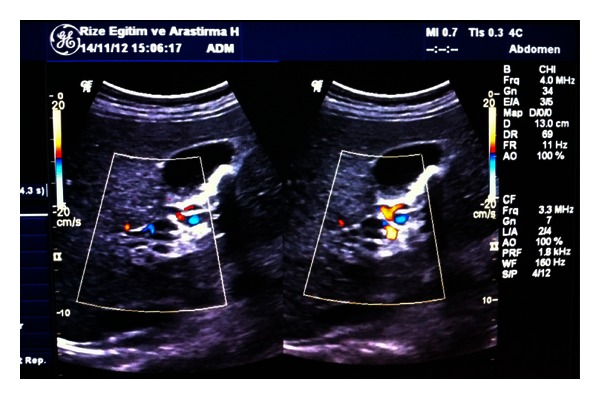
Multiple serpiginous venous collaterals with turbulent venous flow around the thin thrombosed portal vein consistent with cavernous transformation of the portal vein can be seen in the color Doppler ultrasound image.

Three liters of serous ascites was noted within the abdomen during C/S. Bleeding in the form of oozing from suture sites was detected during uterine closure. Hemostasis was maintained by additional sutures. After ascertaining that the uterine tonus was good; the abdomen was closed and a drainage catheter was left in place. Venous oozing was seen at the site of skin sutures as well. The patient was admitted to the intensive care unit for surveillance in case HELLP syndrome or disseminated intravascular coagulation (DIC) syndrome developed. During the postoperative followup the patient had 30 cc/hour continuous serohemorrhagic oozing from the abdominal drain. The patient was transfused with 1 unit of erythrocyte and the hemograms were stable. The oozing became serous during the second postoperative day and gradually decreased. Her vital signs were stable, but she had thrombocytopenia, anemia, and lymphopenia. On the 3rd postoperative day, the patient developed severe abdominal pain, abdominal distention, and fever. The drain catheter started to discharge ascites. A gastroenterology consultation was requested to determine the ascites etiology. Empirical intravenous (IV) antibiotics (cefoperazone-sulbactam) were started for possible peritonitis. The endoscopic examination revealed grade 2 esophageal varices and portal gastropathy. The patient's previous medical records were requested from the other medical facility and they showed that she had suffered from acute PVT 2 years ago. The ultrasound examination of the abdomen showed widespread ascites within the abdominal cavity, chronic portal vein thrombosis and cavernous transformation, splenomegaly, and gallbladder sludge (Figure 1). Thrombotic risk profile was negative for factor V Leiden, prothrombin gene *G20210A*, hyperhomocysteinemia, antithrombin III deficiency, protein C or S deficiency, and antiphospholipid antibodies. Anticoagulation therapy (enoxaparin 6000 anti-Xa IU/0.6 mL/day), diuretics, sodium restriction, and propranolol (a nonselective beta-blocker) were started. The pain gradually disappeared by the third day of treatment and the ascites were under control on the 7th day of treatment. The patient was referred to the gastroenterology clinic for further follow-up and treatment.

## 3. Discussion

In pregnancy, chronic PVT generally presents as repeated episodes of variceal bleeding, asymptomatic splenomegaly or features of hypersplenism and, rarely, as jaundice and ascites [5]. Two large series on pregnant women with chronic PVT revealed that variceal bleeding is the most common clinical complication followed by thrombosis, abdominal pain, jaundice, and incidental splenomegaly [3, 6]. The hypervolemic state of the pregnancy causes an increase in the portal flow, which contributes to high portal pressure that is transmitted to the upper gastrointestinal collateral veins and thus increases the risk of variceal bleeding [7]. The patient presented here had no prepregnancy and post-pregnancy follow-ups regarding PVT. Anemia, thrombocytopenia, abdominal pain and ascites were the most prominent findings. Liver function tests and bilirubin levels were normal and she had no history of hematemesis. We believe that the uncontrolled and untreated state of our patient may have rendered her prone to complications.

Studies have suggested that fertility is near normal among women with chronic PVT as normal liver function is usually maintained in these patients [7, 8]. Women of childbearing age account for approximately 25% of patients with noncirrhotic PVT [3].

There is little data about the outcome of pregnancy on women with chronic PVT which is mostly determined by patients age and the underlying condition causing the thrombosis [9]. Involvement of the superior mesenteric vein appears to be associated with a poorer outcome [9]. Variceal bleeding and hypersplenism are the common manifestations of chronic portal vein thrombosis. Gastric varices are seen in 30–40% of patients with chronic portal vein thrombosis. Pregnancy may be hazardous in these patients as it increases the frequency of variceal bleeding and occasionally the first episode of variceal bleeding may occur during pregnancy [5]. Ideally, women with a history of portal hypertension should undergo preconception endoscopy and prophylactic sclerotherapy if varices are identified. Only 8.6% of patients receiving this treatment experience upper gastrointestinal bleeding in pregnancy [10]. Previous reports suggested variceal bleeding to be particularly frequent, occurring in approximately 15% of pregnancies in patients with noncirrhotic portal hypertension [8]. 

Aggarwal et al. reported outcome of 26 pregnancies in 14 women with chronic portal vein thrombosis [6]. They concluded that pregnancy outcome is expected to be successful in women with extrahepatic portal vein obstruction if the disease is adequately controlled prior to pregnancy; patients who conceived after treatment of esophageal varices or decompression operation had a successful outcome. The major problem faced was hypersplenism leading to thrombocytopenia and anemia.

Hoekstra et al. analyzed the maternal and perinatal outcomes of 45 pregnancies in 24 women with chronic PVT [3]. They showed a favorable outcome in 64% of pregnancies. Pregnancies with an unfavorable outcome were associated with a higher platelet count at diagnosis. Bleeding from esophageal varices occurred in 3 patients during pregnancy, all without adequate primary prophylaxis. Genital or abdominal bleeding occurred postpartum in 4 patients, of which only one was having anticoagulation therapy. Thrombotic events occurred in 2 patients, none related to the lower limbs or mesenteric veins. There were no maternal deaths.

A complication of a PVT pregnancy with early onset severe preeclampsia with HELLP syndrome was reported with a prevalence of 4.3% [3]. Increased blood pressure in this setting may further aggravate the patient's symptoms. Not only pregnant state but the preeclampsia as well may increase the bleeding complications. 

Cavernous transformation of the portal vein is easily diagnosed by sonography since gray scale and color Doppler images fail to demonstrate a normal caliber portal vein in the porta hepatis. Instead, multiple serpentine channels are seen. Color and duplex Doppler confirm the presence of portal venous type flow within those tortuous channels. Associated findings may include esophageal gastric junction, gastric varices, gallbladder wall varices among intra- or extrahepatic biliary tree dilatations.

Labor and vaginal delivery is preferable for the gravida with portal hypertension and varices. A passive second stage of labor may be advisable to avoid due to intra-abdominal and variceal pressure increase [10]. If the abdominal wall varices are cut during cesarean delivery, there is a risk of excessive bleeding. Thrombocytopenia from hypersplenism may also contribute to surgical bleeding. Therefore, if possible, C/S should be avoided.

Anticoagulation therapy is initiated to prevent the extension of thrombosis to superior mesenteric veins and their small branches in patients with chronic portal vein thrombosis. In such patients, intestinal ischemia may be a cause of mortality and morbidity. Early anticoagulation therapy and treatment of underlying causes in these patients can prevent the extension of thrombosis and facilitate recanalization of the thrombosed veins in approximately 30–40% of patients [11]. However, the presence of ascites and splenic vein thrombosis in the negative predictor of recanalization. A nonselective beta blockade can be performed to prevent bleeding from esophageal varices as a form of primary prophylaxis. In the followup, if bleeding occurs from esophageal varices, endoscopic band ligation and sclerotherapy must be performed as a secondary prophylactic measure. Moderate ascites can be controlled by diuretics and sodium restriction. These patients with chronic portal vein thrombosis must be examined periodically for disease progression, esophageal varices and complications of portal hypertension. Also, these patients should be informed about the increased fetal and maternal risks in the recurrent pregnancies. 

In conclusion, noncirrhotic PVT is a very rare pathology in pregnant woman which may cause massive ascites. Cases of preeclampsia with massive ascites should be managed with detailed history and clinical examination using a multidisciplinary approach.
